# Tetramethylpyrazine exerts neuroprotective effects in a mouse model of acute hypobaric hypoxia

**DOI:** 10.3389/fphar.2025.1662389

**Published:** 2025-10-15

**Authors:** Xiong Lan, Wei Zhou, Lei Zhou, Huifang Deng, Pan Shen, Zhijie Bai, Chaoji Huangfu, Ningning Wang, Yue Sun, Chengrong Xiao, Zengchun Ma, Pengfei Zhang, Yue Gao

**Affiliations:** ^1^ School of Pharmacy, Guangdong Pharmaceutical University, Guangzhou, China; ^2^ Beijing Institute of Radiation Medicine, Beijing, China

**Keywords:** hypobaric hypoxia, Tetramethylpyrazine, neuroprotective, cognitive dysfunction, TREK-1

## Abstract

**Introduction:**

Cognitive impairment is a common issue for individuals ascending to high-altitude regions, and there is currently a lack of effective preventive or therapeutic medications. Tetramethylpyrazine (TMP), a small-molecule compound with blood-brain barrier permeability, has shown neuroprotective effects in various neurological disorders. This study aimed to investigate its potential protective role against hypoxia-induced cognitive deficits.

**Methods:**

The neuroprotective effects of TMP were evaluated both *in vivo* and *in vitro*. A simulated high-altitude hypobaric hypoxia mouse model was used to assess survival, cognitive function, cerebral ATP levels, and hippocampal histopathology. *In vitro* studies were conducted to examine hypoxia-induced neuronal death using primary neurons and HT22 cells. Furthermore, mechanistic investigations were performed to identify the molecular target of TMP and its functional impact.

**Results:**

TMP treatment significantly prolonged survival and alleviated cognitive impairment in mice exposed to hypobaric hypoxia. It also elevated cerebral ATP levels and reduced hippocampal cellular edema. *In vitro*, TMP reduced hypoxia-induced neuronal death. Mechanistically, TMP was identified to potentially bind to the ion channel protein KCNK2 (TREK-1) and inhibit TREK-1-mediated current.

**Discussion:**

Our findings demonstrate that TMP provides significant neuroprotection under hypobaric hypoxia conditions. The mechanism is linked, at least in part, to the inhibition of the TREK-1 channel. These results position TMP as a promising therapeutic candidate for preventing or treating high-altitude-induced cognitive dysfunction.

## 1 Introduction

The brain is a highly oxygen-dependent organ, consuming nearly 20% of the body’s total oxygen supply. As a result, it is particularly vulnerable to hypoxia. Under high-altitude hypoxic conditions, cognitive functions such as memory and reasoning become impaired. A three-level meta-analysis of 59 studies (1966–2024) revealed that high-altitude hypoxia leads to cognitive impairment, with long-term memory and perceptual functions being the most affected (Jiang et al., 2025). Additionally, individuals who migrated to high-altitude regions exhibited cognitive impairment and sleep disturbances (Zhao et al., 2024). Furthermore, neuropsychological tests revealed that high-altitude exposure led to cognitive deficits in 69 subjects (Chen et al., 2017). Hypobaric hypoxia also induces morphological changes in the brain. For example, MRI studies revealed that hypoxia reduced gray matter volume in the left putamen, along with decreased regional homogeneity and impaired functional connectivity with other brain regions (Micaux et al., 2025); Brief episodes of hypoxia prompt a translocation of water into the intracellular compartment of the cerebral white matter (Long and Bao, 2022). Animal studies have shown that prolonged and intense exposure to hypoxic or ischemic conditions leads to neuronal cell death in hypoxia-susceptible regions of the brain, such as the cornu ammonis region 1 (CA1), cornu ammonis region 3 (CA3) and the dentate gyrus within the hippocampus, as well as the thalamus, cerebral cortex, and striatum (Snyder et al., 2017; Gavrish et al., 2022). Moreover, hypoxia also leads to a reduction in oxidative phosphorylation in neurons (Aboouf et al., 2023), resulting in decreased ATP production (Li et al., 2023), and consequently, neurons become more reliant on glycolysis for energy supply (Jiang et al., 2025).

Neural activity requires substantial energy expenditure to maintain ion concentration gradients across cell membranes. Under hypoxic conditions, when energy becomes limited, ion channels are activated as an adaptive or detrimental response (Hu et al., 2025; Suganthan et al., 2025). Notably, TREK-1, a two-pore-domain background potassium channel, responds to multiple stimuli and participates in diseases such as depression, epilepsy and stroke. Although an early study revealed that TREK-1 plays a protective role in spinal cord and cerebral ischemia, subsequent studies demonstrated its detrimental role in focal cerebral ischemia (Heurteaux et al., 2004; Zheng et al., 2022). Inhibition of TREK-1 by 3-n-butylphthalide may contribute to neuroprotective effect (Ji et al., 2011). These studies suggest that TREK-1 may also be involved in hypoxic brain injury.

TMP is one of the alkaloid components found in Ligusticum chuanxiong. TMP exhibits multiple pharmacological activities, such as anti-inflammatory, antioxidant, anti-apoptotic, vasodilation and endothelial protection (Du et al., 2021; Li G. et al., 2025; Wang et al., 2025; Xie et al., 2025). As TMP is capable of crossing the blood-brain barrier (Zhang et al., 2025), it exerts neuroprotective effects in experimental models of multiple neurological disorders. For example, TMP protects rats against cerebral ischemia-reperfusion injury via activating PI3K/Akt pathway (Ding et al., 2019); TMP ameliorates cognition of Alzheimer’s disease mice model by inhibiting ubiquitination of somatostatin receptor 4 (SSTR4) (Weng et al., 2021); TMP promotes the repair of spinal cord injury by alleviating ferroptosis (Liu et al., 2024). Recently, we found TMP exerts cardioprotective effects under hypobaric hypoxic condition in mice (Zhang et al., 2024), however, whether TMP is neuroprotective under hypobaric hypoxia is largely elusive.

In this study, we aimed to elucidate the neuroprotective effects of TMP under hypobaric hypoxia conditions and explore the mechanisms underlying its neuroprotective actions. Specifically, we intended to observe the effects of TMP on the survival and cognitive function of mice under hypobaric hypoxia, and to clarify its role in hypobaric hypoxia-induced cerebral pathological alterations. Furthermore, we aimed to explore the impact of TMP on hypoxia-induced neuronal injury *in vitro*, and to clarify the underlying mechanisms through target prediction, molecular docking, interaction validation, and electrophysiological experiments.

## 2 Materials and methods

### 2.1 Animals

C57BL/6 mice were housed in a specific pathogen-free (SPF) environment, and all animal experiments were conducted in compliance with laboratory animal ethics guidelines. In the survival rate assay, 6-week-old mice were exposed to a hypobaric hypoxia environment at 26.4 kPa. 26.4 kPa is the atmospheric pressure at an altitude of 10,000 m. According to the literature, this altitude is frequently used to investigate hypoxia tolerance in mice or rats (Davis et al., 2021; Xu et al., 2021; Wang et al., 2024). In other animal experiments, 6-week-old mice were exposed to a hypobaric hypoxia environment at 35.6 kPa for 12 h 35.6 kPa is the atmospheric pressure at an altitude of 8,000 m. Shuhui Dai et al. reported acute hypobaric hypoxia at 8,000 m causes obvious cognitive deficits in mice (Dai et al., 2024). TMP was purchased from Macklin (T819555, Shanghai, China), dissolved in saline, and administered via oral gavage; Memantine was purchased from MCE (HY-B0591, New Jersey, USA), suspended in saline, and administered via oral gavage; Spadin was purchased from MCE (HY-P1422A, New Jersey, USA), dissolved in saline. Control mice received an equivalent dose of saline via oral gavage or intraperitoneal injection.

### 2.2 Survival rate analysis under hypobaric hypoxia

6-week-old mice were treated with TMP at various doses or memantine at 20 mg/kg by gavage. Then the mice were exposed to a hypobaric hypoxia environment at 26.4 kPa 1 h after administration. The period from the atmospheric pressure reached 26.4 kPa until the death of the mouse was recorded as the survival time of the mouse under extreme hypoxia. The survival curves were plotted using GraphPad Prism 7.

### 2.3 The novel object recognition test

The novel object recognition test is designed to evaluate the ability of mice to recognize and remember newly introduced objects in their environment. Two objects (A and B) of the same shape, size and color were placed in an open field, with A and B positioned 25 cm apart and fixed in parallel. During the habituation phase, the animals are introduced into the open field and allowed to freely explore two identical objects for 10 min. On the second day, the mice were reintroduced into the open field, with object B replaced by a novel object C. The mice were allowed to freely explore two objects for 10 min. The movement trajectory of the mice were recorded and analyzed using SMART V3.0 behavioral tracking and analysis system (Barcelona, Spain). The Novel Object Recognition Index (NOI) was calculated as follows: NOI = Time spent exploring the novel object/(Time spent exploring the novel object + Time spent exploring the familiar object).

### 2.4 The morris water maze test

After receiving two consecutive days of drug administration, the mice were exposed to hypobaric hypoxia for 12 h, followed by the water maze test. The test was conducted using a circular pool (diameter: 1.5 m) filled with opaque water. The water temperature was maintained at 22 °C ± 1 °C. A hidden platform (12 cm diameter) was submerged ∼1 cm below the water surface. Mice’s activities were recorded and analyzed using SMART V3.0 behavioral tracking and analysis system (Barcelona, Spain). On the habituation day, the mice were allowed to freely swim for 60 s without a platform to reduce stress. During the training days (Days 1–5), the circular pool was divided into four quadrants (southeast, southwest, northeast, and northwest) without physical boundaries. The platform was submerged ∼1 cm below the water surface in the southwest quadrant. The mice were released from 5 different start points. Each trial lasted until the mice found the platform or max 60 s. The inter-trial interval was 15–30 min to prevent fatigue. On the test day (Day 6), the platform was removed and each mouse was allowed to swim for 60 s. The time spent in the target quadrant (where the platform was) was used to assess the spatial memory of the mice. During the intervals between the water maze tests, the mice were housed in the hypobaric hypoxia chamber and received a daily drug administration.

### 2.5 ATP measurement

The ATP contents in brain, heart, liver, and muscle tissues were measured using an ATP assay kit (S0026, Beyotime, Shanghai, China) through the proposed method. This kit is developed based on the principle that firefly luciferase requires ATP to provide energy for catalyzing luciferin to produce luminescence. When both firefly luciferase and luciferin are in excess, the luminescence intensity is directly proportional to the ATP concentration within a certain range. Briefly, per 20 mg tissues from indicated group were added with 100 μL of lysis buffer, and then the tissues were homogenized using a tissue homogenizer (AM100, Ants scientifc instruments, Beijing, China). The homogenate was centrifuged at 12,000 × *g* for 5 min and the supernatant was collected for ATP detection. 20 μL of supernatant was added into the well of a black 96-well plate with clear bottom, after which the supernatant was mixed with 100 μL of ATP detection solution per well using a pipette. The relative light units (RLU) was measured immediately using a BioTek imaging system (Cytation5, Winooski, USA). According to the instructions of this kit, the RLU value exhibits a linear relationship with ATP content, so the relative ATP content is equivalent to the relative RLU value, which was determined by normalizing each sample’s RLU value to that of a Control sample.

### 2.6 Lactate measurement

The lactate levels in brain, heart, liver and muscle tissues were measured by a cobas analyzer (c311, Roche, Basel, Switzerland) with the lactate detection kit. Briefly, per 20 mg tissues from indicated group were added with 100 μL of RIPA buffer (50 mM Tris-HCl, 150 mM NaCl, 1% Triton X-100, 1% sodium deoxycholate, 0.1% SDS), and then the tissues were homogenized using a tissue homogenizer (AM100, Ants scientifc instruments, Beijing, China). Then the tissue lysates were centrifuged at 10,000 × *g* for 5 min at 4 °C, and the supernatants were collected. 100 μL of supernatants were pipetted for lactate measurement as per the instrument guidelines.

### 2.7 Histology

After the experiment, mice brain tissues were fixed in 4% paraformaldehyde solution for 48 h. Then the brain tissues were dehydrated through 70%, 80%, 90%, 95%, 100% ethanol solutions. The tissues were cleared in xylene, and then embedded in paraffin wax for 2 h. The embedded tissues were cut into thin sections (4 μm) using a microtome (RM2125 RTS, Leica, Wetzlar, Germany) and the sections were mounted onto glass slides. The slides were immersed in xylene for 10 min to remove paraffin, and then rehydrated through descending alcohols (100%, 95%, 90%, 80%, 70%). The slides were stained in hematoxylin for 3 min and rinsed in distilled water. Then the slides were stained in 1% eosin solution for 1 min and rinsed in distilled water to stop staining. The slides were dehydrated through ascending alcohols (70%, 80%, 90%, 95%, 100%) and cleared in xylene for 5 min. Then the slides were mounted and covered with a coverslip. The slides were examined and imaged under a Nikon microscope (ECLIPSE E200, Tokyo, Japan). The relative cell cross-sectional area (Figure 3B) was calculated by the Photoshop CS6 software. Briefly, open the H&E-stained image in Photoshop CS6, trace neuronal contours with the Lasso Tool, and log the selection area via the Measurement Log panel to record the pixel dimensions. The relative area of each neuron was calculated by normalizing its pixel value to that of a Control neuron. For the relative cell number (Figure 3C), H&E-stained sections from the same hippocampal region were selected. The number of neuronal cells was quantified, and the relative cell number for each section was calculated by normalization to a Control section.

### 2.8 Immunofluorescence (IF) staining

After the experiment, mice brain tissues were fixed, dehydrated, cleared, embedded and cut into sections as mentioned above. The sections were mounted onto glass slides. The slides were immersed in xylene for 10 min to remove paraffin, and then rehydrated through descending alcohols (100%, 95%, 90%, 80%, 70%). Then The slides were subjected to antigen retrieval by microwave heating in 0.01 M citrate buffer (pH 6.0) for 5 min. Then the slides were rinsed in PBS and then treated with 0.3% Triton X-100 in PBS for 8 min to permeabilize. The slides were washed with PBS and incubated with blocking buffer (5% normal goat serum) for 1 h at room temperature. Then the slides were incubated with NeuN antibody (ab104224, abcam, Cambridge, UK, 1:500 dilution) and GFAP antibody (ab7260, abcam, Cambridge, UK, 1:500 dilution) overnight at 4 °C in a humidified chamber. The slides were washed 3 times with PBS and incubated with CoraLite488-conjugated Goat Anti-Mouse IgG (SA00013-1, proteintech, Wuhan, China, 1:1,000 dilution) and CoraLite594-conjugated Goat Anti-Rabbit IgG (SA00013-4, proteintech, Wuhan, China, 1:1,000 dilution) for 1 h at room temperature. Then the slides were washed 3 times with PBS and incubated with DAPI (1 μg/mL in PBS) to label nuclei. Finally, the sections were mounted with coverslips and observed under a fluorescence microscope (Ti-E, Nikon, Tokyo, Japan) for image acquisition. The labelled and double-stained cells were quantified using Photoshop CS6 software.

### 2.9 Isolation of primary mice neuron

Cell culture plates were coated with 10 mg/mL poly-D-lysine (A3890401, Gibco, New York, USA) and incubated at 37 °C for 8 h. Then the plates were washed twice with sterile water and once with PBS, and then dried in a biosafety cabinet for later use. The newborn mice were euthanized and the brain were aseptically dissected under a stereomicroscope. The meninges and blood vessels on the surface of the brain were carefully removed. Then the brains were cut into small pieces and digested with 0.125% trypsin at 37 °C for 10 min. The digestion was terminated by serum and the mixture was filtered through a 70 µm sterile filter mesh to collect the cell suspension. Following centrifugation (500 × *g*, 5 min), the pellet was collected and the cells were resuspended in neuronal growth medium (PM151223, Pricella, Wuhan, China) containing 10% fetal bovine serum and seeded in poly-D-lysine-coated plates. After 12 h of seeding, the serum-containing medium was replaced by serum-free neuronal growth medium containing B-27 (17504044, Gibco, New York, USA).

### 2.10 Cell culture

The primary mice neurons were cultured in neuronal growth medium (PM151223, Pricella, Wuhan, China) containing B-27 (17504044, Gibco, New York, USA). The mouse hippocampal neuronal cell line HT22 cells were cultured in DMEM high-glucose medium (11995-065, Gibco, New York, USA) containing 10% fetal bovine serum (13011-8611, Every green, Zhejiang, China) and 50 U/mL penicillin (P1400, Solarbio, Beijing, China). The normoxic condition used to culture cells was 21% O_2_. For hypoxia treatment, the cells were maintained in a 1% O_2_ environment.

### 2.11 CCK-8 assay

The effect of TMP on the viability of primary neurons and HT22 cells under 1% oxygen was measured using a CCK-8 Cell Proliferation Assay Kit (C0005, TargetMol, Shanghai, China). Primary neurons or HT22 cells were seeded on a 96-well plate at 5,000 cells/well. The primary neurons or HT22 cells were exposed to varying concentrations of TMP at 0, 25, 50, 75, 100, 150, 200 μM, followed by incubation under hypoxic conditions (1% O_2_). The primary neurons were subjected to hypoxic exposure for 72 h while the HT22 cells were exposed to hypoxia for 24 h. Control cells were treated with PBS and cultured in a normoxic incubator. Then 10 μL of CCK-8 reagent was added to 90 μL of culture medium per well. After incubation for 2 h, the absorbance at 450 nm was detected by a BioTek microplate reader (Cytation5, Winooski, USA).

### 2.12 Annexin V-FITC apoptosis assay by flow cytometry

An Annexin V-FITC Apoptosis Detection Kit (AD10, DOJINDO, Kumamoto City, Japan) was used to evaluate the apoptosis in HT22 cells. The HT22 cells were digested with 0.25% trypsin and centrifuged at 500 × *g* for 5 min. The supernatant was removed and the cell pellet was resuspended in 400 µL of 1× Annexin V binding Buffer, after which 5 µL of Annexin V-FITC and 5 µL of Propidium Iodide (PI) were added sequentially in the dark. After mixing thoroughly, the mixture was incubated in the dark for 15 min. The apoptosis rate was measured using a flow cytometer (FACSAria, BD Biosciences, New Jersey, USA). Annexin V-FITC was detected with excitation wavelength at 488 nm and emission wavelength at 530 nm; PI was detected with excitation wavelength at 488 nm and emission wavelength at 616 nm.

### 2.13 LDH measurement

The LDH content in the HT22 cell culture supernatant was measured using an ELISA kit (MM-43732M2, Meimian, Jiangsu, China). Briefly, the cell culture supernatant was collected and added to the wells of a ELISA microplate. After incubation for 1 h at 37 °C, the wells were washed with PBS for 5 times. Then the wells were added with antibody solution (50 µL/well). After incubation for 30 min at 37 °C, the wells were washed with PBS for 5 times and added with substrate solution (50 µL/well). After incubation for 10 min, a stop solution (50 µL/well) was added to the well and the absorbance at 450 nm was detected by a BioTek microplate reader (Cytation5, Winooski, USA). The concentration of LDH was obtained using a standard curve.

### 2.14 Calcein-AM/propidium iodide (PI) double staining

The live and dead HT22 cells and primary neurons were detected by a calcein-AM/PI double staining kit (C542, DOJINDO, Kumamoto City, Japan). Briefly, the cell culture medium was removed and the cells were washed with PBS 2 times. The 100 μL of calcein-AM/PI working solution (2 μM calcein-AM and 1.5 μM PI diluted in PBS) was added to the well of a 96-well plate. The plate was incubated at 37 °C for 15 min. The calcein-AM positive cells (live cells) and PI positive cells (dead cells) were visualized using a BioTek imaging system (Cytation5, Winooski, USA) with 490 nm and 535 nm excitation wavelength.

### 2.15 Molecular docking

First, the structure file of the protein complex crystal in PDB format (PDB ID: 6W8C) was retrieved and downloaded from the RCSB PDB website. This protein structure was processed using Discovery Studio 2019 software, which involved removing water molecules and other ligands. The multimeric protein was then separated into its individual chains as independent structural units, and each was saved in PDB format. The small molecule ligand was energy-minimized using Chem3D 20.0 with the MM2 force field and saved as a PDB file after optimization. Both the protein and ligand PDB files were subsequently processed using AutoDock Tools (including adding hydrogens, assigning charges, and defining atom types) and saved in PDBQT format. Next, a grid box (Grid Box) was constructed centered on the active site of the target protein, with dimensions sufficient to allow conformational sampling of the ligand. The box parameters were recorded and saved in a configuration file (config.txt). The docking parameters were set with an exhaustiveness of 9 and the number of generated conformations set to 500, while all other parameters remained at their default values. Finally, molecular docking between the small molecule ligand and the target protein was performed using AutoDock Vina. The docking run was executed with the command.\vina--config config.txt, which generated the output files. Upon completion, AutoDock Vina produced nine resulting conformations. The software ranked these conformations by analyzing interaction modes, spatial conformations, and a scoring function. The conformation with the lowest theoretical binding free energy was selected for subsequent visual analysis. The binding free energy is typically a negative value, where a higher absolute value indicates a stronger interaction between the ligand and the target protein.

### 2.16 Cellular thermal shift assay (CETSA)

The cellular thermal shift assay was conducted to analyze potential engagement between TMP and TREK-1. HT22 cells were treated with TMP (100 µM) or PBS for 4 h. Then the cells were harvested by trypsinization, centrifuged and resuspended in PBS. Cell suspensions were aliquoted to seven PCR tubes and heated at 44, 47, 50, 53, 56, 59, and 62 °C, respectively for 3 min. Then the samples were frozen in liquid nitrogen to halt denaturation. The cells were lysed by freeze-thaw cycles (3×), centrifuged and the supernatant was collected for Western blot analysis. Briefly, the supernatant was mixed with loading buffer and heated at 100 °C for 10 min. Then the samples were subjected to a SDS-PAGE electrophoresis with voltage at 120 V. The gel was transferred to a PVDF membrane with electric current at 100 mA for 2 h. The membrane were blocked with 5% skim milk for 1 h at 37 °C and then incubated with TREK-1 antibody (47807, Signalway Antibody, Maryland, USA) diluted to 1/1,000 by skim milk for 8 h at 4 °C. Then the membrane was incubated with goat anti-rabbit IgG (ZB-2301, ZSGB-bio, Beijing, China, 1:4,000 dilution) for 1 h at 37 °C and chemiluminescent development was used a chemiluminescent substrate (34579, Thermo Scientific, Massachusetts, USA).

### 2.17 Electrophysiology recording

We used a CHO cell line stably expressing the TREK1 channel, which was constructed by Beijing Innovative CRO + Explorer Biotechnology Co., Ltd. The cells were plated at a density of 8 × 10^3^ cells per coverslip and cultured in a 24-well plate. The patch-clamp procedure was as follows: Borosilicate glass capillaries were pulled into recording pipettes using a micropipette puller. The pipettes were then filled with intracellular solution and mounted onto a pipette holder. The coverslip with cells was placed in a recording chamber on the stage of an inverted microscope. Using a micromanipulator under the inverted microscope, the electrode was immersed in the extracellular solution, and the pipette resistance was recorded. The pipette was then gently advanced to contact the cell surface, and gentle suction was applied to form a seal. Fast capacitance compensation was performed at this point. Further suction was applied to rupture the cell membrane, establishing the whole-cell recording configuration. After achieving the whole-cell configuration, the cell membrane potential was clamped at −80 mV. The voltage was stepped from −80 mV to −100 mV, then ramped to +60 mV over 500 ms. Data were acquired every 5 s to observe the effect of the drug on the TREK-1 current.

### 2.18 Statistical analysis

One-way ANOVA was employed to compare three or more groups. The *post hoc* test used with the one-way ANOVA to evaluate the differences between groups was Dunnett’s multiple comparisons test. Log-rank test was employed to compare the survival rate. GraphPad Prism 7 was used to analyze the data and draw the figures. Data are shown as the mean ± s.d. Error bars represent the standard deviation (s.d.) of the mean. p < 0.05 was considered to indicate a statistically significant difference.

## 3 Results

### 3.1 TMP prolongs the survival time of hypobaric hypoxic mice in a dose-dependent manner

**FIGURE 1:**
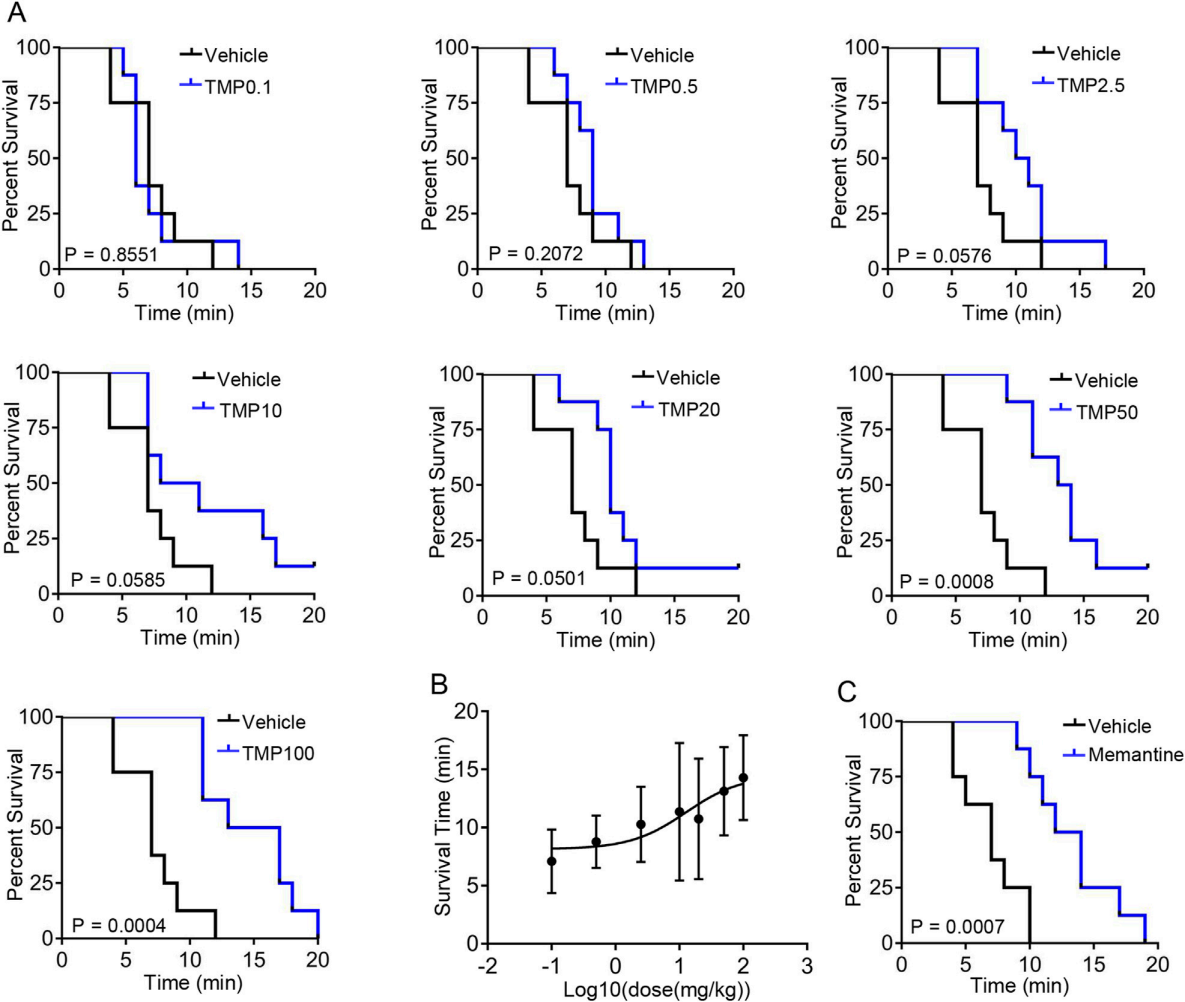
TMP prolongs the survival time of hypobaric hypoxic mice in a dose-dependent manner. **(A,B)** Mice were treated with saline (Vehicle), 0.1 mg/kg TMP (TMP0.1), 0.5 mg/kg TMP (TMP0.5), 2.5 mg/kg TMP (TMP2.5), 10 mg/kg TMP (TMP10), 20 mg/kg TMP (TMP20), 50 mg/kg TMP (TMP50) or 100 mg/kg TMP (TMP100) by gavage. Then the mice were exposed to hypobaric hypoxia (26.4 kPa) 1 h after administration. Survival time was measured from the pressure reached 26.4 kPa until the mice’s death. The survival curves **(A)** and log10 (dose)-survival time curve **(B)** were plotted, n = 8 mice per group. **(C)** Mice were treated with saline (Vehicle) or 20 mg/kg memantine (Memantine) by gavage. Then the mice were exposed to hypobaric hypoxia (26.4 kPa) 1 h after administration. The survival curves were plotted, n = 8 mice per group. Data in **(B)** were shown as the mean ± s.d. Statistical analyses in **(A,C)** were performed with log-rank test. The nonlinear regression curve in **(B)** was plotted using GraphPad Prism 7.

To investigate the neuroprotective role of TMP under hypoxia, we first analyzed whether TMP improved survival in mice exposed to hypobaric hypoxia. Mice were treated with saline (Vehicle), 0.1 mg/kg TMP (TMP0.1), 0.5 mg/kg TMP (TMP0.5), 2.5 mg/kg TMP (TMP2.5), 10 mg/kg TMP (TMP10), 20 mg/kg TMP (TMP20), 50 mg/kg TMP (TMP50) or 100 mg/kg TMP (TMP100) by gavage. Then the mice were exposed to hypobaric hypoxia (26.4 kPa, equivalent to the atmospheric pressure at 10,000 m) 1 h after administration. According to a previous study, TMP reaches its maximum concentration in the brain approximately 38 min after oral administration (Liao et al., 2016). Survival time was measured from the moment the air pressure reached 26.4 kPa until the mice’s death. The TMP50 and TMP100 groups exhibited significantly prolonged survival compared to the vehicle group, as determined by log-rank test (P < 0.001) (Figure 1A). The TMP2.5, TMP10, and TMP20 groups also exhibited improved survival compared to Vehicle controls, but without reaching statistical significance (P > 0.05) (Figure 1A). We found that TMP improved survival time in a dose-dependent manner, approaching its maximal effect at the dose of 50 mg/kg (Figure 1B). The EC50 of TMP was 12.75 mg/kg (Figure 1B). As memantine exhibits neuroprotective effects under hypoxic conditions, we used it as a positive control and found that it also extended the survival time of mice exposed to hypobaric hypoxia (Figure 1C). These results demonstrate that TMP enhances the survival of hypoxic mice in a dose-dependent manner.

### 3.2 TMP alleviates cognitive impairment induced by hypobaric hypoxia in mice

**FIGURE 2:**
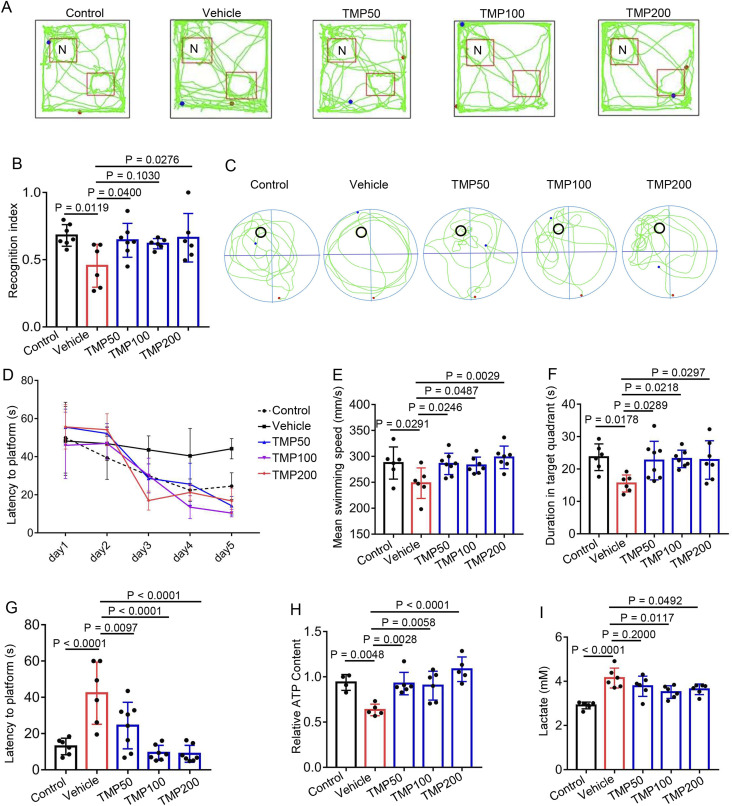
TMP alleviates cognitive impairment induced by hypobaric hypoxia in mice. **(A,B)** The mice were administered normal saline (Vehicle), 50 mg/kg TMP (TMP50), 100 mg/kg TMP (TMP100), or 200 mg/kg TMP (TMP200) via oral gavage once daily for 2 consecutive days. One hour after the final administration, the mice were exposed to a hypobaric hypoxic environment at 35.6 kPa for 12 h. Mice kept in a normal condition for a similar period served as control (Control). Then these mice were subjected to the novel object recognition test. The representative images of mice movement trajectory **(A)** and the recognition index (The ratio of novel object exploration time to total exploration time) **(B)** were shown, n = 6 to 7 mice per group (Control, n = 7; Vehicle, n = 6; TMP50, n = 7; TMP100, n = 6; TMP200, n = 6). **(C–G)** Mice were grouped, administered and exposed to hypobaric hypoxia as described in **(A,B)** then these mice were subjected to the Morris water maze test. The following data were collected: **(C)** The representative images of mice movement trajectory during the probe trial; **(D)** The latency to the platform at day 1–5 during the training session; The mean swimming speed **(E)** duration in target quadrant **(F)** and latency to platform **(G)** during the probe trial, n = 6 to 8 mice per group (Control, n = 6; Vehicle, n = 6; TMP50, n = 8; TMP100, n = 7; TMP200, n = 7). **(H,I)** Mice were grouped, administered and exposed to hypobaric hypoxia as described in **(A,B)**. **(H)** the relative ATP levels of brain tissues, n = 4 to 6 mice per group (Control, n = 4; Vehicle, n = 5; TMP50, n = 6; TMP100, n = 6; TMP200, n = 5). **(I)** The lactate content of brain tissues, n = 4 to 6 mice per group (Control, n = 5; Vehicle, n = 6; TMP50, n = 6; TMP100, n = 6; TMP200, n = 6). Data in **(B,D–I)** were shown as the mean ± s.d. Statistical analyses in **(B,E–I)** were performed with one-way ANOVA with Dunnett’s multiple comparisons test, statistical analyses in **(D)** were performed with two-way ANOVA with Dunnett’s multiple comparisons test. The p-values for the comparisons between groups on days 3, 4 and 5 in **(D)** were all < 0.05.

To elucidate the role of TMP in short-term memory of hypobaric hypoxic mice, we conducted the novel object recognition test. The mice were administered normal saline (Vehicle), 50 mg/kg TMP (TMP50), 100 mg/kg TMP (TMP100), or 200 mg/kg TMP (TMP200) via oral gavage once daily for 2 consecutive days. One hour after the final administration, the mice were exposed to a hypobaric hypoxic environment at 35.6 kPa (equivalent to the atmospheric pressure at 8,000 m) for 12 h. Mice kept in a normal condition for a similar period served as control. The recognition index of the Vehicle group (0.4548 ± 0.1594) was significantly lower than that of the Control group (0.6808 ± 0.08006), indicating the memory of hypobaric hypoxic mice was impaired (Figures 2A,B). The TMP50 and TMP200 groups exhibited significantly higher recognition index than the Vehicle group, demonstrating TMP significantly alleviated memory impairment in hypobaric hypoxic mice (Figures 2A,B).

Next, we conducted the Morris water maze test to evaluate the role of TMP on learning and memory of hypobaric hypoxic mice. Mice were grouped and administered as described above. Compared to the Vehicle group, the TMP50, TMP100 and TMP200 groups exhibited shorter latency to reach the platform on days 3, 4, and 5 of the training session (p < 0.05), demonstrating TMP improved the learning ability in hypobaric hypoxic mice (Figure 2D). We further conducted the probe trail on day 6. Compared to the Vehicle group, all the TMP groups exhibited accelerated swimming speed, increased duration in target quadrant and decreased latency to platform (Figures 2C,E–G), indicating TMP also improved the spatial memory in hypobaric hypoxic mice. We also found that TMP significantly elevated the ATP levels while decreased the lactate levels in hypobaric hypoxic mice brain (Figures 2H,I). Collectively, these results demonstrate TMP alleviates cognitive impairment induced by hypobaric hypoxia in mice.

### 3.3 TMP alleviates hypobaric hypoxia-induced hippocampal cellular edema and astrogliosis

**FIGURE 3:**
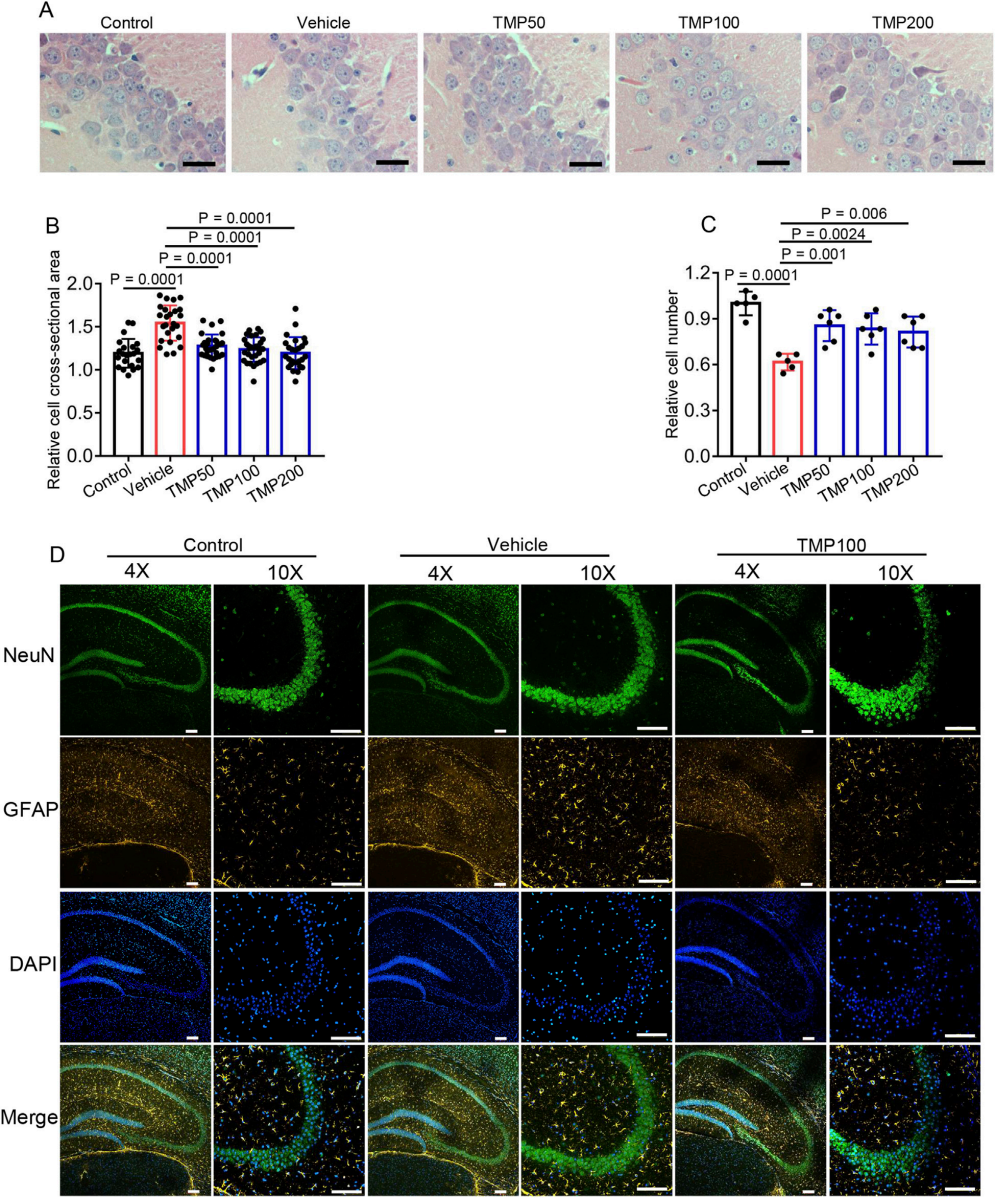
TMP alleviates hypobaric hypoxia-induced hippocampal cellular edema and astrogliosis. The mice were administered with normal saline (Vehicle), 50 mg/kg TMP (TMP50), 100 mg/kg TMP (TMP100), or 200 mg/kg TMP (TMP200) via oral gavage once daily for 2 consecutive days. One hour after the final administration, the mice were exposed to a hypobaric hypoxic environment at 35.6 kPa for 12 h. Mice kept in a normal condition for a similar period served as control (Control). 7 days after hypobaric hypoxic exposure, the mice were euthanized to harvest brain tissues for staining: **(A)** H&E staining images of the hippocampal CA3 region, scale bars: 25 μm; **(B)** The relative cross-sectional area of hippocampal CA3 neurons as mentioned in **(A)** n = 5 mice per group; **(C)** The relative cell number of hippocampal CA3 neurons as mentioned in **(A)** n = 5 to 6 mice per group (Control, n = 5; Vehicle, n = 5; TMP50, n = 6; TMP100, n = 6; TMP200, n = 6); **(D)** Immunofluorescence images of the hippocampal region in mice from the Control, Vehicle, and TMP100 groups, scale bars: 100 µm. Data in **(B,C)** are shown as the mean ± s.d. Statistical analyses in **(B,C)** were performed with one-way ANOVA with Dunnett’s multiple comparisons test.

As the hippocampus is highly sensitive to hypoxia (Zhu et al., 2024), we investigated whether TMP protected against hypobaric hypoxia-induced hippocampal injury. Mice were grouped and administered as described above. 7 days after hypobaric hypoxic exposure, the mice were euthanized to harvest brain tissues for H&E staining. Compared to the Control group (1.193 ± 0.1663), the Vehicle group (1.542 ± 0.2038) exhibited significantly enlarged neurons in hippocampal CA3, demonstrating hypobaric hypoxia induces swelling of hippocampal neurons (Figures 3A,B). All the TMP groups showed reduced size of hippocampal neurons compared to the Vehicle group, indicating TMP relieved hypobaric hypoxia-induced swelling (Figures 3A,B). Furthermore, The TMP groups also exhibited increased CA3 neurons compared to the Vehicle group, demonstrating TMP reduced hypobaric hypoxia-induced neuron loss (Figures 3A,C). Then we performed immunohistochemical staining on the brain sections. Neurons were labeled with the NeuN antibody, and astrocytes were marked with the GFAP antibody. The Vehicle group exhibited increased GFAP-positive cells compared to the Control group, indicating hypobaric hypoxia induced astrogliosis (Figure 3D), which was consistent with previous report (Gavrish et al., 2022). The TMP100 group had decreased GFAP-positive cells compared to the Vehicle group, suggesting TMP alleviated the reactive astrogliosis (Figure 3D). Collectively, these results demonstrate TMP alleviates neuronal swelling, neuronal loss and astrogliosis induced by hypobaric hypoxia.

### 3.4 TMP reduces hypoxia-induced neuronal death *in vitro*

**FIGURE 4:**
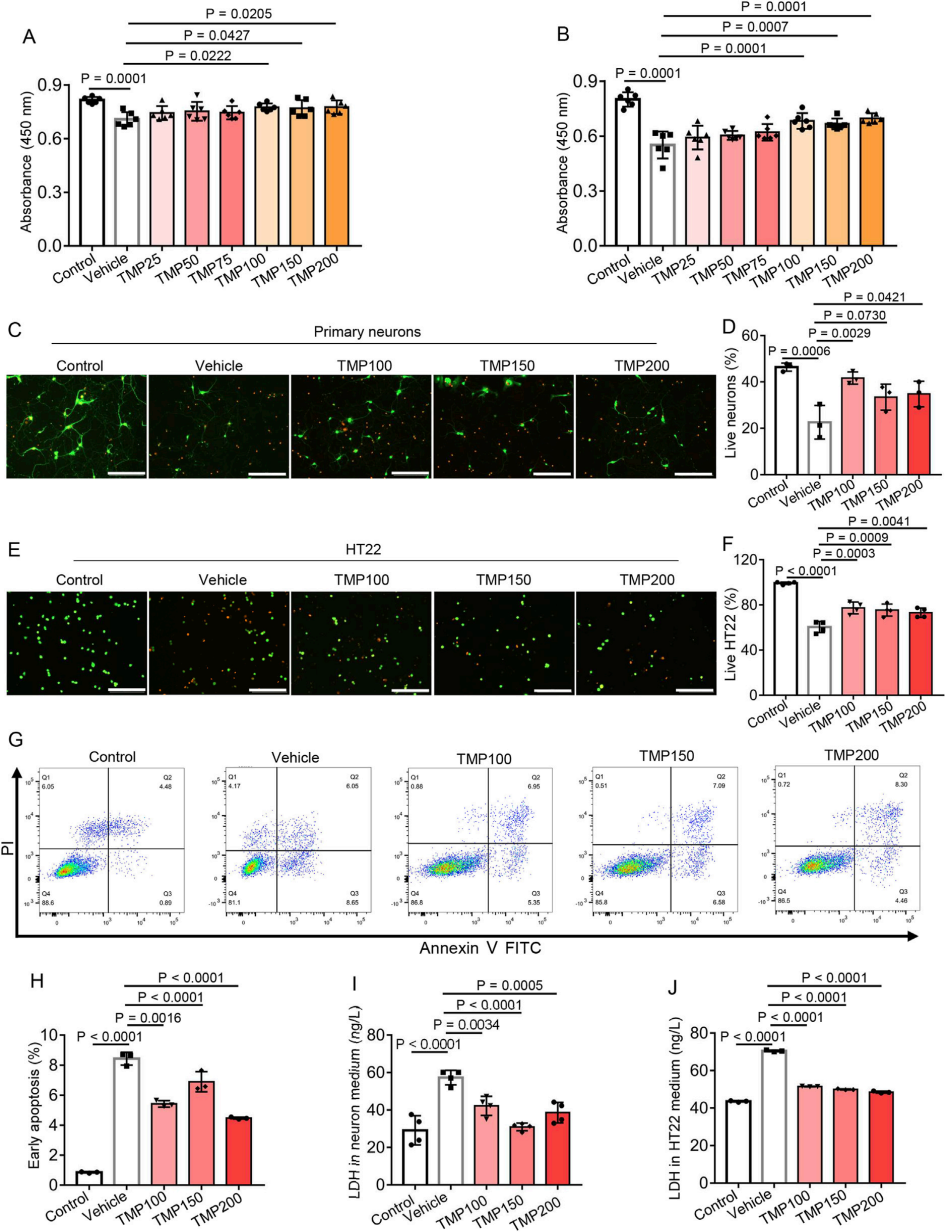
TMP reduces hypoxia-induced neuronal death *in vitro*. **(A,B)** Primary neurons **(A)** or HT22 cells **(B)** were treated with PBS (Vehicle), TMP at doses of 25 μM (TMP25), 50 μM (TMP50), 75 μM (TMP75), 100 μM (TMP100), 150 μM (TMP150) or 200 μM (TMP200), after which the neurons or HT22 cells were subjected to hypoxia (1% O_2_) for 72 h or 24 h, respectively. The control cells (Control) treated with PBS were cultured in normoxic condition. The CCK-8 assay was conducted to assess cell viability, n = 6 per group. **(C–F)** Primary neurons **(C,D)** or HT22 cells **(E,F)** were treated with PBS (Vehicle), TMP at doses of 100 μM (TMP100), 150 μM (TMP150) and 200 μM (TMP200) respectively, after which the cells were subjected to hypoxia (1% O_2_) for 72 h. The control cells (Control) treated with PBS were cultured in normoxic condition. The calcein-AM (green)/PI (red) double staining assay was conducted to assess neuronal viability. The representative images **(C,E)** and proportion of live cells **(D,F)** were shown, scale bars: 200 μm, n = 3 **(D)** or 4 **(F)** per group. **(G,H)** HT22 cells were treated with PBS (Vehicle), TMP at doses of 100 μM (TMP100), 150 μM (TMP150) and 200 μM (TMP200) respectively, after which the HT22 cells were subjected to hypoxia (1% O_2_) for 24 h. The control cells (Control) treated with PBS were cultured in normoxic condition. Representative flow cytometry plot **(C)** and proportion of early apoptotic cells **(D)** was shown, n = 3 per group. **(I,J)** Primary neurons or HT22 cells were treated and subjected to hypoxia as described in **(C–F)** then the LDH content in the cell culture supernatants was measured using an ELISA kit, n = 3 **(J)** or 4 **(I)** per group. Data are shown as the mean ± s.d. Statistical analyses were performed with one-way ANOVA with Dunnett’s multiple comparisons test.

We further investigated the direct effect of TMP on primary neurons and HT22 neuronal cells cultured in 1% O_2_ condition. Primary neurons or HT22 cells were treated with PBS (Vehicle), TMP at doses of 25 μM (TMP25), 50 μM (TMP50), 75 μM (TMP75), 100 μM (TMP100), 150 μM (TMP150), 200 μM (TMP200) respectively, after which the cells were subjected to hypoxia. The control cells treated with PBS were cultured in normoxic condition. The CCK-8 assay was conducted to assess cell viability. Compared to the Vehicle group, The TMP100, TMP150 and TMP200 groups showed elevated absorbance in both primary neurons and HT22 cells, indicating TMP increased neuronal viability under hypoxia (Figures 4A,B). Then we conducted calcein-AM/PI double staining of primary neurons and HT22 cells respectively. Hypoxia decreased the percentage of live cells (green) in both primary neurons and HT22 cells (Vehicle vs Control, Figures 4C–F). TMP treatment significantly increased the percentage of live cells in both primary neurons and HT22 cells compared to Vehicle treatment (Figures 4C–F), demonstrating TMP promoted cell survival under hypoxia.

Next we detected apoptosis of HT22 cells by flow cytometry. The early apoptotic cells were annexin V positive and propidium iodide (PI) negative. Compared to the Control group (0.8533% ± 0.04726%), the Vehicle group (8.433% ± 0.4283%) showed increased percentage of early apoptotic HT22 cell, suggesting hypoxia induced apoptosis of HT22 cells (Figures 4G,H). The TMP100 (5.42% ± 0.2138%), TMP150 (6.893% ± 0.6676%) and TMP200 (4.47% ± 0.06557%) groups showed reduced early apoptosis compared to the Vehicle group (8.433% ± 0.4283%), indicating TMP reduced hypoxia-induced apoptosis of HT22 cells (Figures 4G,H). We further detected the lactate dehydrogenase (LDH) content in primary neuronal and HT22 culture supernatant, which is an indicator of cell injury (Davis et al., 2021). Hypoxia increased LDH content in the supernatants of both cell cultures (Vehicle vs Control, Figures 4I,J). TMP treatment significantly decreased the LDH content compared to Vehicle treatment (Figures 4I,J), indicating TMP alleviated hypoxia-induced cell injury. Collectively, these results demonstrate TMP increases neuronal viability and alleviates neuronal death under hypoxia.

### 3.5 TREK-1 is a potential target of TMP

**FIGURE 5:**
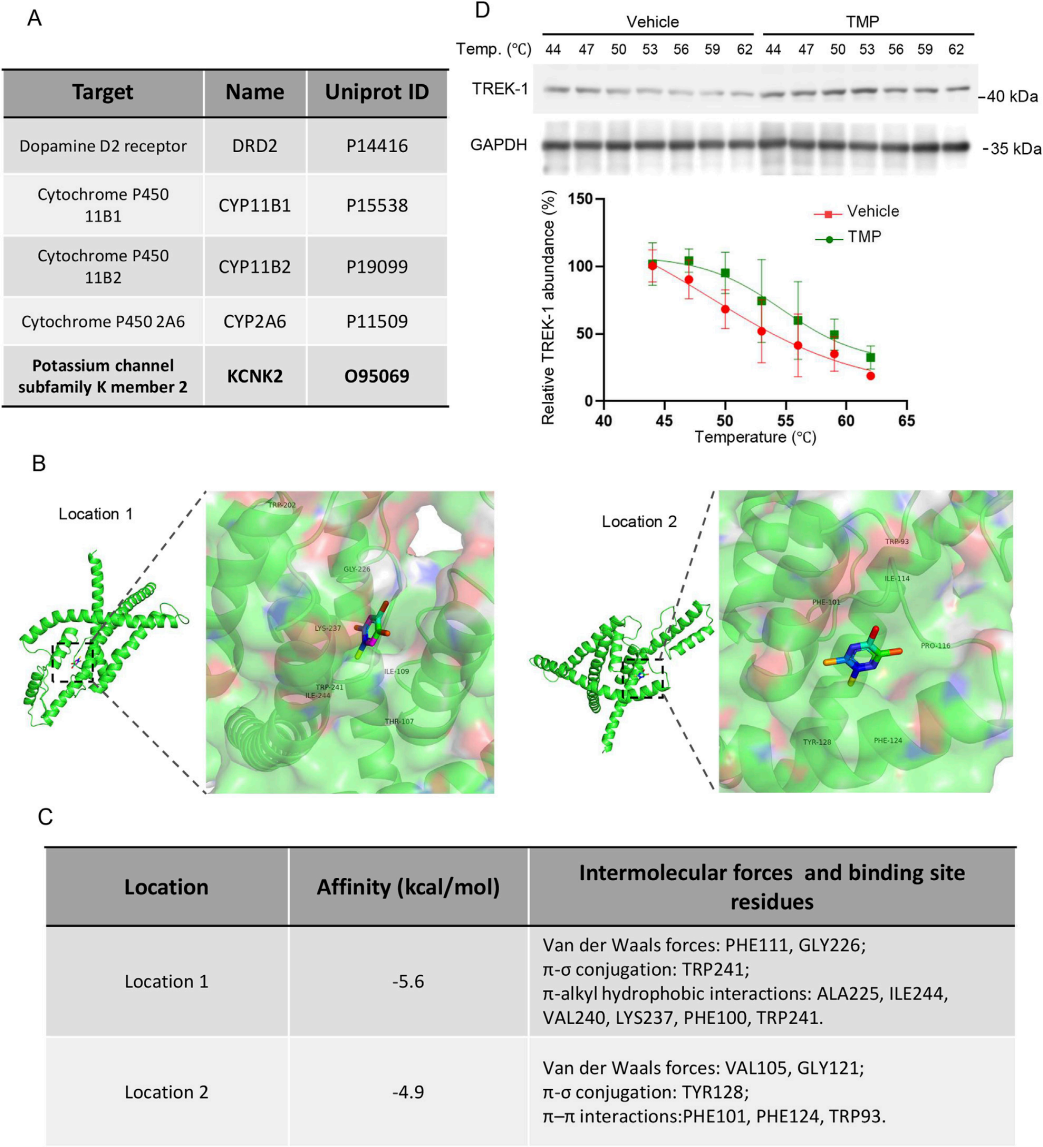
TREK-1 is a potential target of TMP. **(A)** The top-ranked protein predicted to bind with TMP. **(B)** The representative images of molecular docking between TMP and TREK-1 **(C)** The affinity constants, intermolecular forces, and binding residues of TMP and TREK-1 at position 1 and position 2. **(D)** HT22 cells were treated with PBS (Vehicle) or TMP (100 µM) for 4 h, then the cellular thermal shift assay were conducted. The representative images of TREK-1 protein levels and relative TREK-1 abundance were shown. n = 3. Data in **(D)** are shown as the mean ± s.d.

To elucidate the mechanism by which TMP exerted its neuroprotective effects under hypoxic conditions, we predicted potential targets of TMP using the SwissTargetPrediction website (http://www.swisstargetprediction.ch/). Among the top five predicted targets (Figure 5A), dopamine receptor D2 (DRD2) is implicated in brain cognition (Yang et al., 2025b), but it is primarily expressed in putamen rather than hippocampus (Hukema et al., 2025); cytochrome P450 family 11 subfamily B member 1 (CYP11B1) is mainly involved in the biosynthesis of adrenal corticoids (Janot et al., 2025); cytochrome P450 family 11 subfamily B member 2 (CYP11B2) catalyzes the biosynthesis of aldosterone, and is involved in blood pressure regulation, arterial hypertension, and the development of heart failure (Azizi et al., 2025); cytochrome P450 family 2 subfamily A member 6 (CYP2A6) exhibits a high coumarin 7-hydroxylase activity and acts in the hydroxylation of drugs (Tzoupis et al., 2024). KCNK2, also known as TREK-1, has been reported to participate in cerebral ischemia and express widely in the brain. Thus we hypothesized that TMP might protect neurons partially by modulating TREK-1. The binding energies of TREK-1 and TMP at binding site 1 and binding site 2 were −5.6 kcal/mol and −4.9 kcal/mol, respectively (Figures 5B,C). TMP interacts with the corresponding residues of TREK-1 through van der Waals forces, π-σ conjugation, π–π interactions and π-alkyl hydrophobic interactions (Figures 5B,C). To provide additional evidence for the TREK-1-TMP interaction, we conducted a cellular thermal shift assay (CETSA). Compared to Vehicle treatment, TMP treatment increased the thermal stability of TREK-1 protein in HT22 cells, indicating an interaction between TMP and TREK-1 (Figure 5D). These results demonstrate TREK-1 is a potential target of TMP.

### 3.6 TMP inhibits TREK-1 channel

**FIGURE 6:**
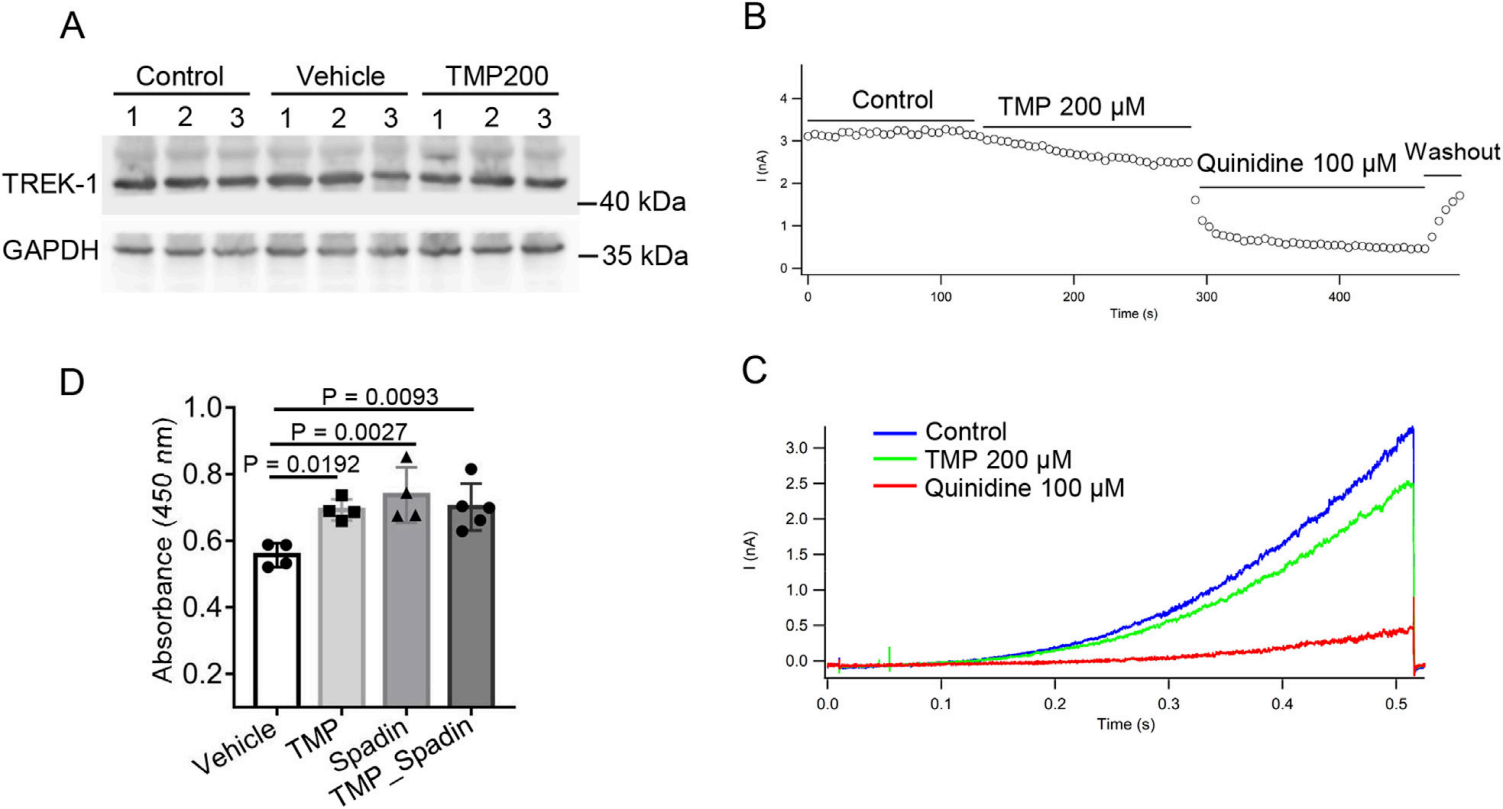
TMP inhibits TREK-1 channel. **(A)** The mice were administered with normal saline (Vehicle) or 200 mg/kg TMP (TMP200) via oral gavage once daily for 2 consecutive days, and then were exposed to a hypobaric hypoxic environment at 35.6 kPa for 12 h. Normoxic mice administered with normal saline served as Control. TREK-1 protein levels of mice brain tissues were detected by Western blot. **(B)** Peak current at different time points after treatment with PBS (Control), 200 μM TMP or 100 μM Quinidine. **(C)** TREK-1 currents recorded by whole-cell patch clamp following PBS (Control), 200 μM TMP or 100 μM Quinidine treatments. **(D)** HT22 cells were treated with PBS (Vehicle), 200 μM TMP (TMP), 100 μM spadin (Spadin) or both (TMP_Spadin), then the cells were subjected to hypoxia (1% O_2_) for 24 h. The CCK-8 assay was conducted to assess cell viability, n = 4 to 5 per group (Vehicle, n = 4; TMP, n = 4; Spadin, n = 4; TMP_Spadin, n = 5). Data in **(D)** are shown as the mean ± s.d. Statistical analyses were performed with one-way ANOVA with Dunnett’s multiple comparisons test.

To investigate the mechanism by which TMP regulates TREK-1, we first measured the protein levels of TREK-1 in the brain tissues of normoxic mice (Control), hypobaric hypoxic mice treated with PBS (Vehicle), and hypobaric hypoxic mice treated with 200 mg/kg TMP (TMP200). TMP did not exert a significant effect on TREK-1 protein levels (Figure 6A). To investigate a potential direct modulation of the TREK-1-mediated potassium leak current by TMP, we performed electrophysiological recordings on CHO cells expressing TREK-1 channels. We applied a voltage step from −80 mV to −100 mV, and then a voltage ramp to +60 mV lasting 500 ms. Compared to the Control group, TMP inhibited the TREK-1-mediated current by 23.71% (Figures 6B,C). Quinidine, served as a positive control, potently suppressed the current (Figures 6B,C). We subsequently used the CCK-8 assay to assess the effects of Vehicle, TMP, spadin, and a combination of TMP and spadin on the viability of hypoxic HT22 cells. Compared to the Vehicle group, spadin significantly increased the viability of hypoxic cells, indicating that inhibiting TREK-1 promoted cell survival under hypoxia (Figure 6D). However, compared to spadin treatment alone, the combination of TMP and spadin did not further enhance cell viability, suggesting that TMP exerted its protective effects against hypoxia by acting on TREK-1 (Figure 6D). These findings suggest that TMP exerts its protective effects against hypoxia by inhibiting the TREK-1 channel.

## 4 Discussion

Acute high-altitude hypoxia impairs cognitive function and triggers symptoms of acute mountain sickness, such as headaches, nausea, and insomnia (Wang et al., 2022; Zidan et al., 2025). In severe cases, it may lead to high-altitude cerebral edema (HACE), posing a significant health risk to travelers or workers in high-altitude regions (Li Z. et al., 2025). However, there remains a lack of effective preventive or therapeutic drugs for hypoxia-induced brain injury. We simulated a high-altitude environment using a hypobaric hypoxia chamber and found that TMP exhibits neuroprotective effects under hypoxic conditions. The neuroprotective effects of TMP are manifested as follows: 1. TMP significantly prolonged the survival rate of mice under hypoxia; 2. TMP ameliorated hypobaric hypoxia-induced cognitive impairment in mice; 3. TMP markedly increased ATP levels in the brains of hypobaric hypoxic mice; 4. TMP alleviated hippocampal cell swelling and glial hyperplasia in hypobaric hypoxic mice; 5. TMP significantly reduced hypoxia-induced neuronal cell death and enhanced neuronal viability *in vitro*.

Hypoxia or ischemia modifies ion channel states, altering membrane potential and disrupting electrical signaling. For example, in hippocampal and dorsal vagal neurons, hypoxia induces the opening of ATP-sensitive K^+^ channels (KATP), leading to potassium efflux and transient neuronal hyperpolarization, which may reduce hypoxia-induced damage by decreasing neuronal excitability (Szeto et al., 2018). Sustained hypoxia may induce depolarization in hippocampal neurons by inhibiting voltage-gated potassium channels or enhancing sodium influx, which may result in neuron damage (Hernansanz-Agustin, 2025); The physical and functional coupling between the ion channel transient receptor potential melastatin-2 (TRPM2) and NMDA receptor (NMDAR) exaggerates NMDAR-induced excitotoxicity during ischemic injury (Zong et al., 2022). Some hypoxia-tolerant animals, such as fish, amphibians, and reptiles, reduce energy demand by modulating ion channel activity (e.g., KATP and NMDAR), thereby maintaining cellular homeostasis (Nagy-Watson and Jonz, 2025; Suganthan et al., 2025). These findings demonstrate that ion channels can exert both protective and detrimental effects on neurons during hypoxia.

TREK-1, a member of the two-pore domain potassium channel family, is expressed throughout the central nervous system (Yang et al., 2025a). TREK-1 activation is triggered by membrane tension, arachidonic acid and lysophospholipids (Bechard et al., 2024; Sorum et al., 2024; Escobedo and Rasband, 2025). In this study we found that inhibiting TREK-1 under hypoxic conditions might exert a neuroprotective effect, as the TREK-1 inhibitor spadin increased the viability of hypoxic cells. However, we acknowledge that using spadin as the sole tool compound for TREK-1 specificity has its limitations. Future studies should utilize genetic models, such as TREK-1 knockout animals, to confirm its role in hypoxia.

Our study lacks direct pharmacokinetic data for TMP in brain tissue at the tested doses. Thus we estimated brain TMP concentration extrapolated from previous pharmacokinetic data and predicted the potential binding of TMP to TREK-1 *in vivo*. According to the Gibbs free energy equation describing the relationship between binding energy (ΔG) and dissociation constant (K_d_) (Gilson and Zhou, 2007):

$$K_{d}=e^{-\Delta G/\text{RT}}$$

where: ΔG = binding free energy (kcal/mol), R = gas constant (1.987 × 10^−3^ kcal·K^−1^·mol^−1^), T = absolute temperature (K), K_d_ = dissociation constant (M). By inputting the ΔG (−5.6 kcal/mol) and T (37 °C, 310 K) into the Gibbs equation, we calculated a K_d_ value of 112 μM. According to Weiguo Liao et al., a single oral dose of 4 mg/kg TMP achieved a brain dialysate concentration of 900 ng/mL, i.e., 6.6 μM (Liao et al., 2016). Therefore, if the brain concentration of TMP is proportional to its oral gavage dosage, a 50 mg/kg gavage dose of TMP is predicted to yield a brain concentration of 82.5 μM, which is close to the K_d_ value. Thus, predictive modeling indicates a possible binding interaction between TMP and TREK-1 *in vivo*. However, we acknowledge that the extrapolated intracerebral concentration of TMP is not precise. Therefore, direct measurement of its brain concentration at the administered dose is necessary in the future.

TMP enhances the survival of hypoxic mice in a dose-dependent manner, with its effect plateauing at 50 mg/kg (Figure 1B), suggesting that 50 mg/kg represents the optimal effective dose for this model. Therefore, given the potential side effects of TMP (Lin et al., 2022), the higher doses (100 mg/kg and 200 mg/kg) used in this study were not required, since 50 mg/kg was already sufficient to achieve near-maximal efficacy.

While our study focused on the neuroprotective effects of TMP in acute hypoxia, the cognitive deficits caused by chronic hypoxia are equally important. Therefore, future studies should explore the efficacy of TMP under chronic hypoxic conditions.

In addition, TMP is a small-molecule compound that may interact with multiple intracellular proteins, suggesting that TREK-1 is likely just one of its target proteins. Therefore, TMP might also exert its neuroprotective effects through other proteins under hypoxic conditions.

In this study, 21% O_2_ was used as the normoxic condition for *in vitro* experiments. While this is commonly used for conventional cell culture, it is substantially higher than the physiological oxygen level in brain tissue (approximately 5%–6%). Therefore, strictly speaking, 21% O_2_ should be considered hyperoxic for neurons. Future studies will consider adopting culture conditions that more closely mimic physiological oxygen levels to better simulate the *in vivo* environment.

In conclusion, our study demonstrated that TMP exhibits neuroprotective effects under hypobaric hypoxia conditions, suggesting its potential to alleviate high-altitude-induced cognitive decline.

## Data Availability

The original contributions presented in the study are included in the article/supplementary material, further inquiries can be directed to the corresponding authors.
